# The highly sensitive determination of serotonin by using gold nanoparticles (Au NPs) with a localized surface plasmon resonance (LSPR) absorption wavelength in the visible region†

**DOI:** 10.1039/d0ra05271j

**Published:** 2020-08-20

**Authors:** Phuong Que Tran Do, Vu Thi Huong, Nguyen Tran Truc Phuong, Thi-Hiep Nguyen, Hanh Kieu Thi Ta, Heongkyu Ju, Thang Bach Phan, Viet-Duc Phung, Kieu The Loan Trinh, Nhu Hoa Thi Tran

**Affiliations:** Faculty of Materials Science and Technology, University of Science Ho Chi Minh City Viet Nam ttnhoa@hcmus.edu.vn; Vietnam National University Ho Chi Minh City Viet Nam; Tissue Engineering and Regenerative Medicine Laboratory, Department of Biomedical Engineering, International University Ho Chi Minh City Viet Nam; Center for Innovative Materials and Architectures (INOMAR) Ho Chi Minh City Viet Nam; Department of Nano-Physics, Gachon University Seongnam-si Gyeonggi-do 13120 Republic of Korea; Laboratory of Advanced Materials, University of Science Ho Chi Minh City Viet Nam; Future Materials and Devices Laboratory, Institute of Fundamental and Applied Sciences, Duy Tan University Ho Chi Minh City 700000 Viet Nam; Faculty of Environmental and Chemical Engineering, Duy Tan University Da Nang 550000 Viet Nam; Department of Industrial Environmental Engineering, College of Industrial Environmental Engineering, Gachon University Seongnam-si Gyeonggi-do 13120 Republic of Korea

## Abstract

The development of improved methods for the synthesis of monodisperse gold nanoparticles (Au NPs) is of high priority because they can be used as substrates for surface-enhanced Raman scattering (SERS) applications relating to biological lipids. Herein, Au NPs have been successfully synthesized *via* a seed-mediated growth method. The LSPR peak is controlled *via* adjusting the gold nanoseed component, and different fabrication methods were studied to establish the effect of sonication time on NP size. The simple, facile, and room-temperature method is based on a conventional ultrasonic bath, which leads to ultrasonic energy effects on the size and morphology of the Au NPs. This research offers new opportunities for the production of highly monodispersed spherical Au NPs without the use of a magnetic stirrer method, as evidenced by ultraviolet-visible reflectance spectra and scanning electron microscopy (SEM) analysis. SEM images indicate that the spherical Au NP colloidal particles are stable and reliable, which paves the way for their use as a nanostructured biosensor platform that can be exploited for multiple applications, for example, in materials science, sensing, catalysis, medicine, food safety, biomedicine, *etc.* The highest enhancement factor that could be achieved in terms of the SERS enhancement activity of these Au NP arrays was determined using 10^−9^ M serotonin (5-hydroxytryptamine, 5-HT) as the Raman probe molecules.

## Abstract 2

The development of improved methods for the synthesis of monodisperse gold nanoparticles (Au NPs) is of high priority because they can be used as substrates for surface-enhanced Raman scattering (SERS) applications relating to biological lipids.

## Graphical abstract

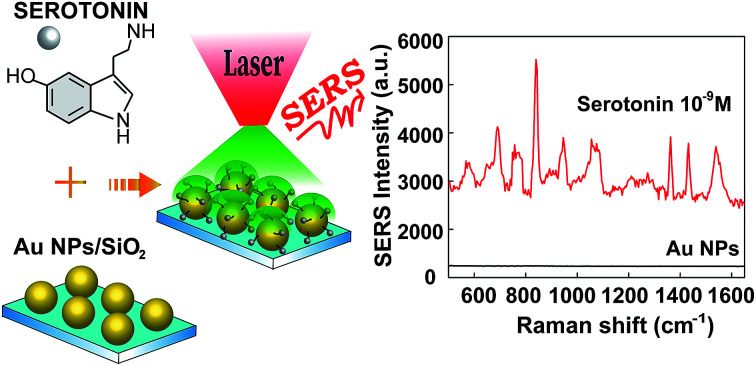

## Introduction

Plasmonics is a term that relates to the localization and manipulation of an electromagnetic wave that propagates along a metal–dielectric interface. For plasmonic applications, the most attractive property of metal NPs is electromagnetic resonance based on the collective oscillation of free electrons, which results in localized surface plasmons (LSPs). LSPs occur when the metal NPs are much smaller than the incident light wavelength (*R* ≪ *λ*).^1^ The metal NPs can serve as antennas to convert light into localized electric fields (E-fields). There are two kinds of models of plasmonic nanostructures: (1) localized surface plasmons (LSPs) and (2) propagating surface plasmons (PSPs). With LSPs, the oscillation can be in resonance with the incident light at a specific excitation frequency (*w*), resulting in the strong oscillation of the surface electrons, which is called a localized surface plasmon resonance (LSPR) mode.^2^ In recent years, LSPR sensors based on metal nanostructures or nanoparticles have generated increasing interest.^3^ In contrast to propagating SPR, LSPR sensors can be fabricated *via* immobilizing metalized nanostructured materials on a substrate (clean glass, optical fiber, *etc.*).^4^ Recently, the decoration of plasmonic nanostructures, such as Au NPs and Ag NPs, has been used to purposefully control the density of the electric field on the surface of the particles. Changes in the oscillation frequency of the electrons could lead to changes in optical properties, such as absorption and scattering, accordingly^5^ and, as a consequence, amplify the electromagnetic field enhancement around the nanoscale metallic particles in the vicinity of their surface. Promising candidates to act as good plasmonic materials have an optical absorption spectrum with a maximum at the LSPR frequency wavelength, which for colloid dispersions containing Au NPs is centered at 520 nm.

The physical properties of LSPR are reported to make it suitable for more sensitive, stable, and selective detection than traditional bulk-metal thin-film prism-based SPR sensors, due to the nanoscale size of the metal particles.^6^ Of all the metallic nanoparticles, Au NPs have been demonstrated to have greatly interesting characteristics for an LSPR shift (spectral shift) that is useful for biosensing applications due to their unique optical properties.^7^ Au NPs are capable of absorbing and scattering light at intensities up to 5 times those of conventional color molecules.^8^ Moreover, the outstanding properties of Au NPs include their non-toxicity, stable structure, high biological compatibility, and easy activation of surfaces for binding to biological molecules and drug molecules through the thiol (–SH) group.^9^ With these unique properties, biomedical applications of gold nanostructures are increasingly being developed, such as sensing and biosensing,^10,11^ imaging,^12^ environmental monitoring, and drug screening.^13^ Remarkably, LSPR sensing strategies based on the modulation of wavelength have been extensively explored and have many observable advantages compared to other sensing modalities. Metallic NP LSPR does not suffer from photo-bleaching, making it superior to conventional fluorophores such as organic dyes.^14^ Comparing plasmonics based on metals (Cu, Ag, Au, Pd, Ru, *etc.*), Au NPs have attractive characteristic LSPR properties, presenting the collective oscillation of free electrons after excitation by electromagnetic incident light.^15^ The positions and shapes of these Au NP plasmon LSPR frequencies are strongly dependent on the size, shape, elemental composition, and interparticle spacing of the NPs, which are responsible for the specific colors of NPs.^16^ Au NPs that are 10–70 nm in size exhibit an LSPR band centered at 525 nm.^17,18^

Many synthesis methods have been developed in an attempt to produce nano-sized Au NPs, such as irradiation methods using different irradiation sources such as microwaves,^19^ gamma rays,^20^ and lasers;^21^ electrochemical methods;^22,23^ and one-step seed-mediated methods using reducing agents such as chemical- and bio-reductants. Abdelghany *et al.* reported an Au NP synthesis method, producing Au NPs with a size of 11–14 nm and a plasmon peak at 531 nm, utilizing γ-irradiation.^24^ Even though this method has some advantages, such as controllable size, high purity, and no reducing agent use for the formation of Au NPs, it also has several disadvantages, including the need for modern and complex equipment, and the low worldwide availability of and restricted access to γ-irradiation. A green method was utilized by Zheng *et al.* for the preparation of Au NPs with a particle size of 25 ± 7 nm using natural biomaterial egg shell membrane (ESM), an eco-friendly and non-toxic biological material.^25^ In order to form Au NPs, ESM was immersed directly into HAuCl_4_ solution without any reducing agent, along with careful pH control to adjust the particle size and control the complex preparation process for synthesizing Au NPs. Turkevich *et al.* developed a method using the same reducing agent both during the seed-mediated growth phase and after to form the desired Au NPs that has many advantages, such as the simple, cost-efficient synthesis procedure.^26^ The procurement of an appropriate reductant has been studied by many different groups with various aims and objectives. Firstly, green reduction, utilizing organic sources such as plant and animal extracts, has been used to reduce the pollution caused by potentially harmful reductants, which is also the biggest weakness of the reduction method. Wei *et al.*^27^ prepared chitosan as a reductant and surfactant for the synthesis of gold and silver nanoparticles, and Hu *et al.*^28^ designed an AgCl@Au photocatalyst with a strong SPR effect from the Ag NPs and a large impact from the surfactant effect. However, there are many drawbacks relating to uneven distributions and the related stabilities of composite photocatalysts during further experiments. Different works have addressed the surfactant effect using sugar and bio-polymers as different reducing agents (chitosan, starch, and sugars),^29^ showing great results for Ag NPs but causing thermodynamic instability due to the low pH of the Au NP solution during the synthesis process, although this benefits the reduction of the Au salt. Besides, other extracts from fungi, fungal microflora, bacteria, and microalgae, including enzymes, proteins, pigments, sugars, and biological macromolecules, have been used in a wide range of nano-metal synthesis procedures, showing utility in the pharmacy field.^30^ However, bio-extracts always struggle from high rates of contamination, leading to particular experimental difficulties and questions about the exact efficiencies of these reductants over other ingredients for synthesis procedures relating to advanced research and future studies. On the other hand, research has pointed out the outstanding advantages of chemical reductants in terms of stabilization and the ability to control particle size depending on the concentration of the reducing agent. Results showed that tetrakis(hydroxymethyl)phosphonium chloride (THPC) could be used to synthesize ultra-nano-size Janus Au NPs that were 2 nm in size, but this approach was limited by the solubility in polar solvents.^31^ Organic acids such as aspartic acid,^32^ tannic acid,^33^ and lactic acid^34^ were considered as prospective reductants for the synthesis of nano-spherical core structures with nano-oriented tubes outside. A series of other chemical reductants, like metal complexes, salts, liposomes, and polymers, were used in different studies and diverse results were obtained.^35^ In studies using starch and glucose by Engelbrekt *et al.*^36^ and fungus extract by Kitching *et al.*^37^ in an attempt to find an outstanding reductant for chemical synthesis procedures, the cost and speed benefits were described. However, some difficulties were seen from the nanoparticle characterization, mostly the wide-range and hard-to-control plasmon peak due to the low pH and thermodynamically unstable synthesis process. Besides, the requirement for uncontaminated experimental and storage areas is also a defect for small laboratories. Therefore, this emphasizes the important point of selecting a suitable reductant according to the future application goals. For medium nanoparticle size stabilization requirements and to obtain a maximum absorbance spectrum wavelength able to respond to a specific signal in the visible region for optical biosensor applications, sodium citrate is always a potential candidate. In this work, control over the physical properties of shape, size, and dispersion were achieved completely based on the effects of citrate-stabilized spherical Au NP growth.

Au NPs show various types of structure and morphology at the nanoscale level, such as rods, wires, tubes, hexagons, spheres, triangles, and irregular shapes. The LSPR absorption band is affected by the particle size, shape, and structure.^38^ According to Mie theory,^39^ small spherical Au NPs exhibit only one plasmon resonance frequency peak, wherever anisotropic particles exhibit two or more plasmon bands based on their shape, which correspond to electric dipole oscillations on the surface.^40^

LSPR-based plasmonic biosensors have emerged as a potential tool for biosensing applications involving noble metal nanoparticles. Many synthesis approaches have been developed, and the best technology is rapidly becoming nanotechnology to incorporate new methodologies for the fabrication of Au NPs for effective sensing using LSPR nanosensors. In this study, a seed-mediated growth method presents a simple and reproducible procedure for the successful production of mono-disperse spherical Au NPs. Spherical Au NPs with a mean particle diameter of 84 ± 0.21 nm were formed when higher gold nanoseed concentrations (3.377 mL) were used. The aggregation state of the Au NPs has an effect on their optical properties. A combination of optical property studies, X-ray diffraction, UV-vis spectroscopy, IR analysis, and electron microscopy analysis was used to follow the crystal growth process, and to determine the absorption plasmon peaks, sizes, and shapes of the Au NPs. Layers of amino-functionalized spherical Au NPs grafted on a glass substrate were probed *via* Fourier-transform infrared spectroscopy and contact angle studies.

The resolution of conventional Raman spectra is based on a relatively weak signal; this has been overcome through the invention of SERS for signal enhancement.^41^ The SERS effect on metal nanoparticles, which is surface-sensitive, is based on interactions (evaluated *via* absorption/extinction spectra) between the incoming light, the target sample molecule, and the metallic surface.^42^ It has been found that the SERS intensity rises as the size of the Ag NPs becomes larger (∼50 nm). However, a further increase in particle size (100–130 nm) leads to lower intensity SERS signals.^43^ Metal colloid nanoparticle aggregates have traditionally been the primary form of SERS-active substrate. Metal nanoparticles (Au and Ag NPs) are considered as common SERS substrates, with enhancement coefficients of up to 10^6^ and above,^44^ because they are not Raman active.^45^ As SERS has some outstanding advantages, such as high sensitivity for chemical and biological molecules, spectral selectivity, and analysis speed,^46^ it has been explored extensively for sensing and imaging applications.^47^

Heterodimers (Au and Ag NPs) have been widely utilized as SERS substrates, and they are good candidates for bio-applications such as the detection of small molecules, drug molecules, and environmental toxins.^48^ In this study, we prepared Au NPs in aqueous solution, and then the SERS activity toward as-prepared serotonin was systematically investigated. Serotonin is the most important human neurotransmitter,^49^ regulating numerous biological processes related to physiological areas, such as cardiovascular function, bladder control, and adaptation to stressors; it plays a role in the pathophysiology of mood disorders;^50^ and it can be used in the diagnosis of diseases such as abnormal blood pressure, kidney disease, and depression.^51^ Several methods have been reported for measuring serotonin, such as high-performance liquid chromatography (HPLC),^52^ the enzyme-linked immunosorbent assay (ELISA),^53^ and SERS.^54^ HPLC has been widely used for serotonin determination in blood. It is a high-efficiency and high-sensitivity detection method but there are some drawbacks, as the procedure is complicated, with no universal detector and the need for time-consuming sample preparation.^55^ The use of a larger number of samples to validate specific biomarkers is required for the measurement of serotonin using the ELISA method.^56^ Among the aforementioned methods, SERS is an analytical method that can be used for the detection of low amounts of serotonin analytes; it is a highly sensitive platform with good stability and reproducibility that is based on exploiting SERS active materials such as metal nanoparticles (*e.g.*, Au and Ag).^57^

In this study, we focus primarily on the SERS performance of as-prepared large-area and uniform Au NP film, which was systematically investigated using Raman probe molecules. Under optimum conditions, 40 min of ultrasound was applied to investigate the activity of Au NPs as SERS-active substrates. SERS signals were obtained at concentrations ranging from 10^−5^ M to 10^−9^ M 5-HT. A serotonin concentration as low as 10^−9^ M was detected, which demonstrates the superior capability of SERS for detecting sub-physiological levels of serotonin. The Au NPs were successfully applied to SERS-based immunoassays.

## Experimental

### Chemicals and reagents

All the chemicals used in this article were of analytical grade. Gold(iii) chloride trihydrate (HAuCl_4_·3H_2_O, 99.9%), sodium citrate tribasic dihydrate (HOC(COONa)(CH_2_COONa)_2_·2H_2_O, 99.0%), and (3-aminopropyl)trimethoxysilane (H_2_N(CH_2_)_3_Si(OCH_3_)_3_, APTMS, 97%) were provided by Sigma-Aldrich Co., Ltd (USA). Sodium hydroxide (NaOH, 96%) was provided by Guangdong Guanghua Sci-Tech Co., Ltd (China). Deionized water (DI, resistivity: 18 MΩ cm, Thermo Scientific Easypure II, Göteborg, Sweden) was synthesized in the Center for Innovative Materials and Architectures (INOMAR), Vietnam National University, Ho Chi Minh City (VNUHCM). Hydrogen peroxide (H_2_O_2_, 30%) was obtained from Merck KGaA (Germany). Sulfuric acid (H_2_SO_4_, 96%) was acquired from Acros Organics (USA). Ethanol (C_2_H_5_OH, EtOH, 99.8%) and methanol (CH_3_OH, 99.8%) were procured from Fisher Ltd (UK). Microscope glass slides (soda-lime glass, chemical composition: SiO_2_ = 75 ± 5 wt%; Na_2_O = 15 ± 2 wt%; CaO + MgO = 10 ± 2 wt%) were purchased from ISOLAB Laborgeräte GmbH, Eschau, Germany.

### Preparation of gold nanoseeds

Gold nanoseed nucleation was performed using sodium citrate tribasic dehydrate (Na_3_Ctr) as a reducing agent and gold(iii) chloride trihydrate (HAuCl_4_·3H_2_O) as a gold precursor. A flask containing 100 mL of 1 mM HAuCl_4_ and 200 μL of 1 M NaOH equipped with a condenser was heated up to 100 °C while undergoing gentle mixing with a stir bar at 250 rpm. Then, 10 mL of 38.8 M Na_3_Ctr was quickly added. A visible color change of the Au NP solution to a wine-red color occurred, which takes around 2 min from the time of addition, and the reaction was kept stable for 15 min more before shutting it down and cooling down to room temperature (RT). Finally, DI water was added to make 100 mL of final seed solution, which was stored avoiding sunlight and in a refrigerator at 4 °C.

### The seeded growth synthesis of Au NPs

The seed-mediated growth procedure in our work includes the following steps. Citrate-capped Au NPs were synthesized based on the described method. 277 μL of 1 mM HAuCl_4_ was added to 10 mL of 38.8 mM sodium citrate, Au nanoseeds, and deionized water (DI) water at room temperature, and growth proceeded *via* two synthesis methods.

#### Method A: the seeded growth synthesis of uniform Au NPs using a magnetic stirrer

As introduced by Kimling and co-workers,^58^ the use of a magnetic stirrer for fabricating Au NPs has been intensively explored during the Turkevich method. A mixed reagent solution is stirred using a Teflon-coated magnetic bar. The 40 mL of total solution that was stirred included 3.377 mL of Au NP seeds, 227 μL of 1 mM HAuCl_4_, 176 μL of 38.8 mM Na_3_Ctr, and 36.22 mL of DI water, and stirring occurred at a speed of 250 rpm at room temperature. The final Au NP solution was covered with aluminum foil to prevent the effects of photoreduction and was stored in glass serum vials at 4 °C.

#### Method B: the conventional ultrasonic bath synthesis of highly dispersed Au NPs

The steps in method B are similar to those used in method A, but an ultrasonic bath approach replaced Au NP dispersion in the liquid under magnetic stirring. An ultrasonic bath (Elmasonic EASY 60H, 220–240 V, Germany) was used for the mixing step with the same volumes of ingredients. The particular effects of ultrasound on chemical synthesis have been investigated since a study by Richards and Loomis.^59^ In the ultrasonic-assisted synthesis of Au NPs, the powerful ultrasonic energy approach leads to bond cleavage, and it affects the size distribution of the particles in solution more than a conventional heating method because of the penetration properties of ultrasonic irradiation through solution. The size of the nanoparticles is critically dependent on the ultrasonic energy generated at 180 W of actual power input and a frequency of 40 kHz, or on the sonication reaction time. It is important to adjust the bath temperature carefully *via* refilling with water, as long-term use can cause the temperature to rise to more than 40 °C, which is harmful both to the ultrasonic bath itself and the Au NP solution. High temperature during growth may cause the Au NPs to show low absorbance without changing the maximum absorbance wavelength. The mixing time in both methods was varied to 10, 20, 30, and 40 min, and the resulting wine-red Au NP solutions were stored at 4 °C in a dark environment in a refrigerator to avoid nanoparticle aggregation and the effects of photoreduction.

### AuNP staining of an amino-capped glass substrate

Microscope glass slides were soaked in 0.5% NaOH for 15 min and then washed using 0.5% NaOH in an ultrasonic bath for 10 min. The glass substrates were washed using distilled water, ethanol, and acetone for 10 min each time. Finally, the glass slides were cleaned using methanol-soaked cotton and dried naturally.

**Fig. 1:**
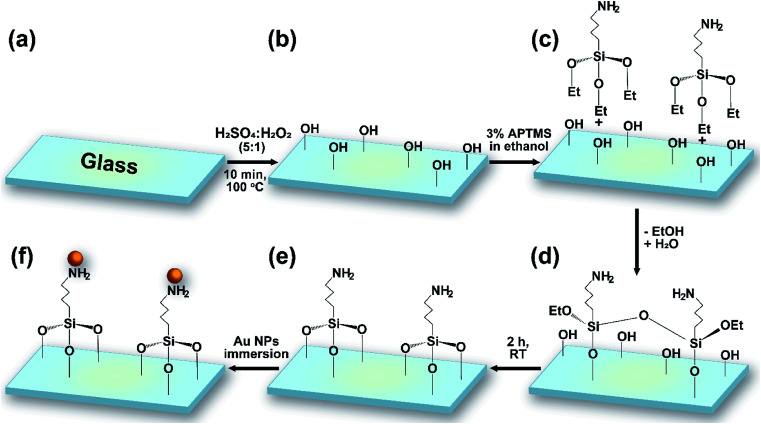
A schematic illustration of the overall stages of the surface functionalization of a glass slide with Au NPs: (a) bare glass; (b) the glass surface with hydroxyl groups, functionalized using piranha solution; (c) APTMS silanization; (d) reduction and Si–O formation; (e) NH_2_-functionalized glass; and (f) Au NP functionalization.

The glass substrates and silicon wafers were cut into 1 × 1 cm pieces and cleaned following a procedure including several washing steps and solvent treatment with DI, piranha solution, methanol, and hydrogen fluoride (HF). Reactive hydroxyl groups were generated through shaking the wafers in piranha solution (H_2_SO_4_/H_2_O_2_ = 5 : 1 v/v) for 10 min. After washing with water for 5 min, the substrates were dried naturally at room temperature. 3% APTMS was grafted *via* immersing the wafers in a solution of silane in EtOH at room temperature for 2 h. Samples were washed twice, sonicated for 15 min in EtOH, and heated at 80 °C for 1 h. The modified substrates were finally immersed in gold colloidal solution for numerous lengths of time (4, 12, 16, and 20 h) (Fig. 1). The resulting gold-coated substrates were stored in a humidity-controlled desiccator chamber.

### Preparation of SERS substrates

5-HT was used as the probe molecule for Raman detection. Droplets of Au NP solution (20 μL) were dropped onto the glass substrate *via* a spin-coating method. 20 μL of 5-HT at different concentrations (ranging from 10^−5^ to 10^−9^ M) was immediately dropped onto the substrate in the same places as the Au NP droplets. The samples were analyzed *via* Raman spectroscopy after drying at room temperature. Raman measurements were performed using a Horiba XploRa One spectrometer with an excitation laser wavelength of 532 nm. A silicon wafer (520 cm^−1^) was used to calibrate the instrument.

### UV-visible absorption spectroscopy characterization

The preliminary characterization of the kinetics, final colloid stability, and formation of Au NPs after various reaction times was conducted using UV-visible spectroscopy after diluting the colloid Au NPs with deionized water 20 times (DI was used for all measurements as a blank to adjust the baseline solution). UV-vis spectra were obtained using a V-730 visible/NIR (wavelength range: 200 to 1100 nm) spectrophotometer (JASCO, Tokyo, Japan). A UV quartz cuvette (5 mm optical path length) was filled with a standard 2 mL of Au NP solution.

### Fourier-transform infrared spectroscopy (FT-IR) analysis

FT-IR is used as a standard technique to confirm the formation of characteristic groups and optical components, such as hydroxyl groups using piranha solution, the addition of amino sites using APTMS, and the crosslinking of the glass substrate, with the automatic setting-up and checking of measurement parameters. The results were recorded using a Frontier FT-IR spectrometer from Bruker Vertex 70, Germany. The measurements were performed at room temperature in the wavenumber range of 4000–400 cm^−1^.

### Powder X-ray diffraction (PXRD)

PXRD analysis indicates the crystallinity of a desolvated material. A Bruker D8 Advance diffractometer with Ni-filtered Cu Kα (*λ* = 1.54178 Å) radiation operating at 40 kV and 40 mA (1600 W) was used.

### Scanning electron microscopy (SEM)

The surface morphologies of substrates covered with Au NPs were examined using a scanning electron microscope (JSM-IT100, JEOL, Japan) at a voltage of 5 kV. Images were taken at different scales and in different regions (scale bars corresponding to 100 nm and 1 μm) of clean glass covered with the colloidal Au NPs.

### Contact angle (*θ*) measurements

Static water contact angles were measured on a glass window and on hydroxyl- and amino-modified glass at room temperature using an automatically controlled drop volume followed by axisymmetric drop shape analysis *via* Image J software. The instrument (Phoenix 300, Surface Electro Optics), which uses a CCD camera for the measurement of surface tension and image analysis processing software, was purchased from Tustin, USA. The water (Milli-Q system, 18.2 MΩ cm) droplet volume was 0.5 μL and *θ* was recorded 3 s after the drop was deposited on the substrate. The coefficients of variation of the contact angles were reported based on the averages of results obtained from five tests.

### Raman spectroscopy

Raman spectra were obtained using a confocal microprobe Raman system, HORIBA XploRA One (HORIBA Scientific, HORIBA Ltd., HORIBA Europe GmbH), with a holographic notch filter and liquid-nitrogen-cooled CCD detector, working with a laser wavelength of 532 nm (green light). To improve the important parameter of signal-to-noise ratio, the solution irradiation time was set to 60 accumulations of 10 s at each sampling position. Raman spectra were compiled with laser power at the sample of 1 mW, because although increased laser power would increase the band intensities, it would also destroy the sample. All SERS experiments were performed at room temperature.

## Results and discussion

### Preparation of gold nanoseed solutions

**Fig. 2:**
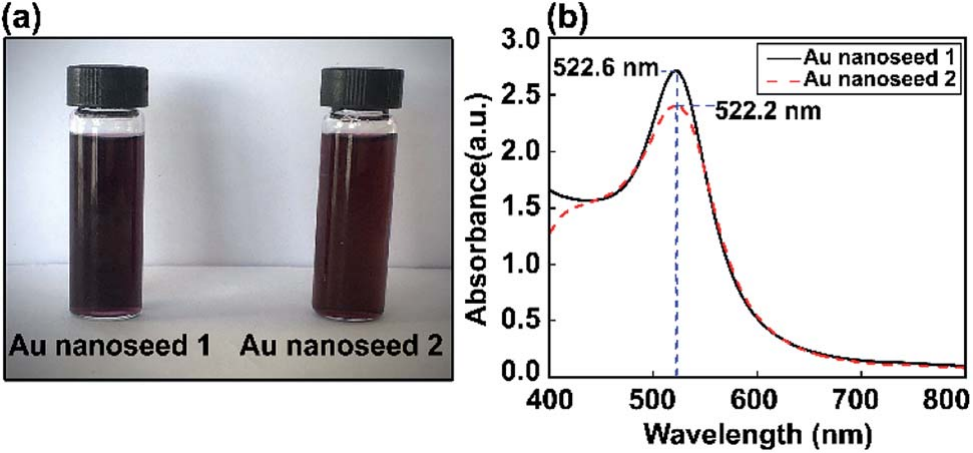
(a) Photographs of two Au nanoseed solution samples and (b) corresponding UV-vis absorption spectra.

Au nanoseed preparation is described above. Fig. 2 shows a photograph of the Au nanoseeds and UV-visible spectra. A shift in wavelength to 522.6 nm was observed for one sample and a second sample showed a single LSPR peak at 522.2 nm. The stability of the Au nanoseeds was also checked and they were stable for several weeks with no sign of aggregation during storage under the right conditions. The optical properties showed the monodispersity of the Au nanoseeds, with a well-defined optical extinction band located at 522.2 nm from a single Au NP plasmon mode. For isotropic spherical nanoparticles, the existence of only one attenuation peak, which corresponds to the excitation of a single plasmonic mode, is proposed.

### Synthesis of Au nanoparticles using a magnetic stirrer

**Fig. 3:**
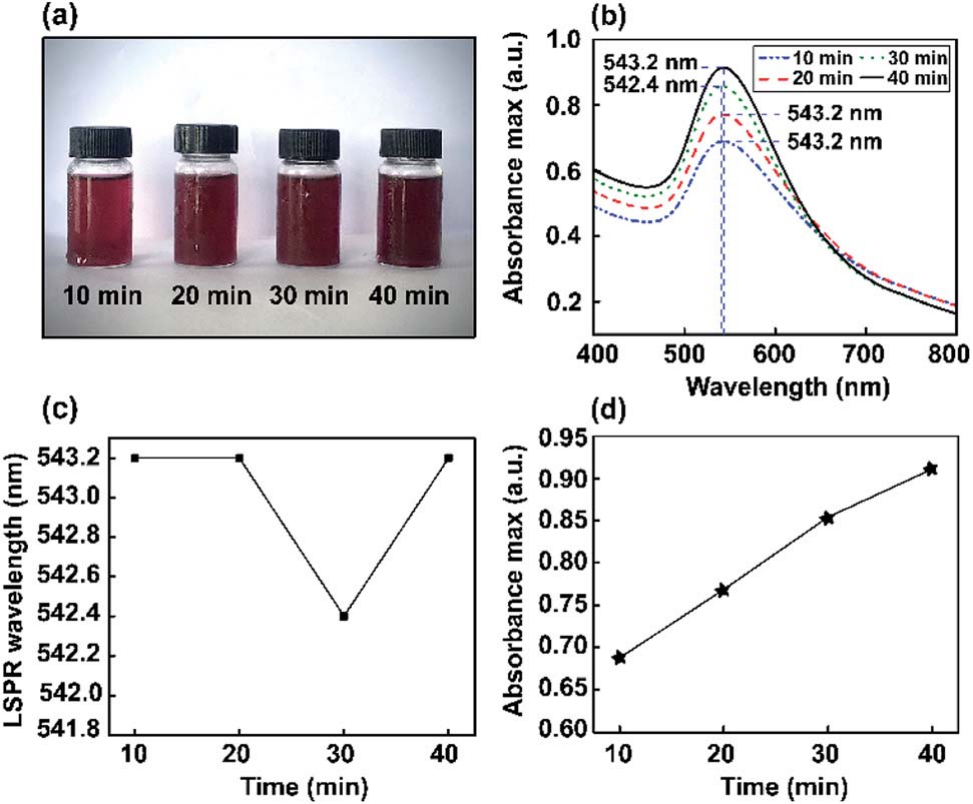
The study of Au NPs obtained *via* the magnetic stirring method. (a) Digital photos showing the colors of the dispersions. (b) UV-vis absorbance spectra. (c) LSPR wavelength (nm). (d) The absorbance maximum of Au NPs formed using various stirring times (10, 20, 30, and 40 min).

Color changes were observed and the corresponding optical absorbance of Au NP solutions was analyzed *via* a UV-visible spectrophotometer. The UV-visible spectra of Au NPs as a function of stirring time are shown in Fig. 3. The changes in color intensity of the NPs were monitored using a soda-lime glass bottle and UV-visible spectrophotometer. In the UV-vis spectrum, a narrow resonance plasmon band was present as an absorption peak in the visible region of 542–543 nm. Thus, different stirring times (10, 20, 30, and 40 min) were investigated to study the constant maximum absorption intensity LSPR peak of the Au NPs. It was found that ions may induce the aggregation of NPs in solution. However, this method did not significantly interfere with optimum conditions for the absorbance of Au nanoparticles being obtained at 543 nm.

Furthermore, corresponding photographs of the prepared Au NPs obtained using different stirring times (10, 20, 30, and 40 min) are presented in Fig. 3a. The color of the solution gradually becomes darker than the Au nanoseeds, because of the different size distributions of the high-yield Au NPs.

### Ultrasonic synthesis of highly dispersed Au nanoparticles

**Fig. 4:**
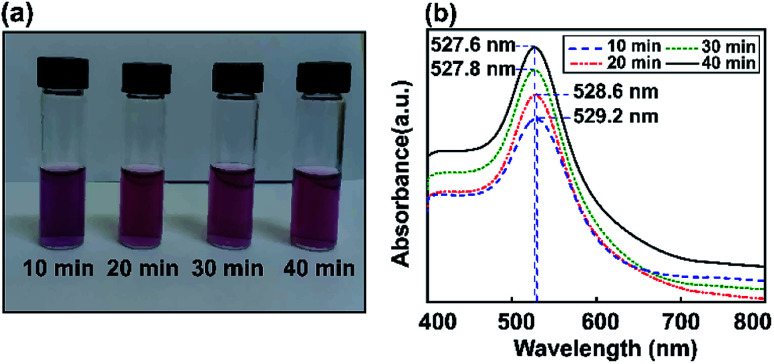
The study of Au NPs obtained *via* the ultrasonic method. (a) Digital photos showing the colors of the dispersions. (b) Absorbance spectra for Au nanoparticles (NPs) obtained after various ultrasonic times (10, 20, 30, and 40 min).

Fig. 4 shows experimentally measured optical extinction spectra highlighting the effects of ultrasonic energy on the size of Au NPs when controlling the sonication time (10, 20, 30, and 40 min). The formation of Au NPs can also be monitored by recording the plasmonic curves during the reaction. *Via* synthesizing Au NPs using different ultrasonic exposure times, the surface plasmon resonance can easily be tuned to give an absorbance maximum at around 527.6 nm. The LSPR spectrum of Au NP solution after 40 min of ultrasonic reaction is shifted to a lower absorption maximum plasmon wavelength compared to the above results using the stirring method. This shows the tunable optical properties of gold using the ultrasonic irradiation method, which decreases the size of the particles and reduces the extinction coefficient as well.

Based on the Mie model and the resolution of the Maxwell equations,^60^ many studies have pointed out the best absorption range for each nanometal size to get the best optical performance depending on the applications. In the case of Au NPs for biosensors, SPR is clearly visible based on a maximum absorbance wavelength in the UV-vis range of 520–540 nm.^61,62^

In the one-step seed-mediated synthesis of Au nanoseeds, the size of spherical non-aggregated Au NPs can be estimated to change based on elements such as the concentration of precursor salt, the nanoseed growth time, *etc.* Based on the ultrasonic method investigated for different reaction times (10, 20, 30, and 40 min), it is shown that the maximum wavelength of the resulting Au NPs in the visible spectral region tends to decrease as the growth time increases. No visible Au NP solution color change was observed as the LSPR peak moved from 527.6 nm to 527.8 nm during the synthesis of Au NPs, due to the SPR, and the absorption edge at a shorter wavelength was thought to be due to interband transitions of d-band electrons.^63^ Thus, the maximum absorbance wavelength was almost saturated after an optimal sonication time of 40 min using the ultrasonic bath method, also obtaining the required wavelength for the LSPR effect. Subsequently, we use a reaction time of 40 min as the preferred conditions to obtain Au NPs for further experiments. As a temporary conclusion, the amplification of the local field due to LSPR is still expected at 527 nm for all these samples. In conclusion, we have proved that this modified seed-mediated growth technique is suitable for producing consistent Au NPs. The formation of Au NP solutions with a LSPR absorption peak position at 527.6 nm can occur through a nucleation and growth process involving Au nanoseeds, in which the nucleation to growth rate ratio and reaction time give the final size of the nanoparticles.

**Fig. 5:**
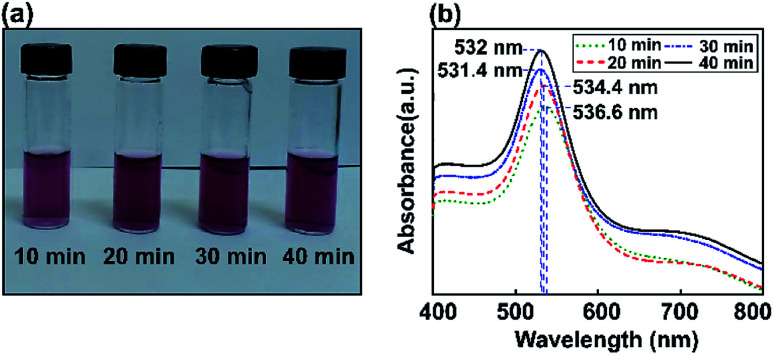
UV-Vis spectrometry analysis of Au NP formation at a lower precursor concentration of 0.811 mL (ultrasound exposure time: 10, 20, 30, and 40 min). (a) Digital images of the samples, and (b) UV-vis spectra of the reaction mixtures.

The influence of Au NP size on surface plasmon resonance is illustrated in Fig. 5, where the saturated absorption maximum is between 531.4 and 536.6 nm. After the Au NP seed amount was decreased from 3.377 mL to 0.811 mL, the Au NP plasmonics showed a significant broadening of the peak at 532 nm. The intensity of the LSPR peak increased with a decrease in the gold precursor concentration. An increase in LSPR peak intensity at a lower precursor concentration is attributed to an increase in the formation of Au NPs.^64^ A minor red shift in the SPR wavelength from 531.4 to 536.6 nm was observed when the precursor concentration was decreased from 3.377 mL to 0.811 mL and various ultrasonic exposure times were used (10, 20, 30, and 40 min), indicating the augmentation of the mean particle diameter of the synthesized Au NPs.

Comparing the proposed magnetic stirrer and ultrasonic synthesis methods for obtaining Au NPs for LSPR, the potential of the ultrasound-based method was seen based on the LSPR peak at 527.6 nm.

### The self-assembled orientation of amino (–NH) groups on the silicon substrate surface

Silicon wafers (resistivity: 0.0011 Ω cm; 1 × 1 cm^2^; Siltronix, France) were immersed in piranha solution for 10 min to decorate the glass substrate with hydroxyl groups. Amine-terminated surfaces were generated using 3% APTMS in EtOH. Surface IR, in ATR mode for the silicon substrates, was used to characterize the molecular composition of the modified glass substrates.

**Fig. 6:**
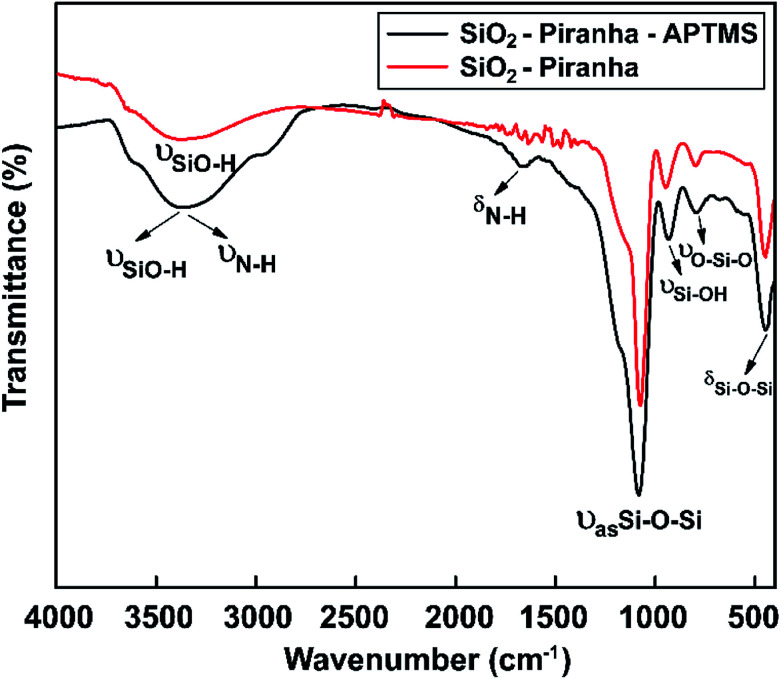
FT-IR spectra showing the surface chemical modifications of samples obtained *via* piranha solution and APTMS in the range of 4000 to 450 cm^−1^.

As a result, for the APTES-modified sample, the characteristic IR bands that were expected (–NH_2_ groups) were observed (Fig. 6). The FTIR spectrum of an APTMS-functionalized silicon wafer is shown in Fig. 6; at around 3390 cm^−1^, a symmetric hydrogen-bonded SiO–H stretching mode corresponding to water absorption is seen.^65^ Moreover, the absorption band at 3400 cm^−1^ can be assigned to symmetric N–H stretching upon the modification of the silicon surface with APTMS.^66^ The FTIR curve also has a new vibrational mode at around at 1650 cm^−1^, which is the bending vibration absorption peak of N–H.^67^ In the spectral region between 1200 and 480 cm^−1^, the band at 1080 cm^−1^ is due to the asymmetric stretching vibration of Si–O–Si, the band at 447 cm^−1^ confirms the existence of aromatic symmetric Si–O–Si bending,^68^ the band at 932 cm^−1^ is from Si–OH stretching,^69^ and the band at 794 cm^−1^ is from O–Si–O stretching. The appearance of the above-mentioned absorption bands proves that hydroxyl groups and amine groups were successfully grafted onto the silicon wafer.

**Fig. 7:**
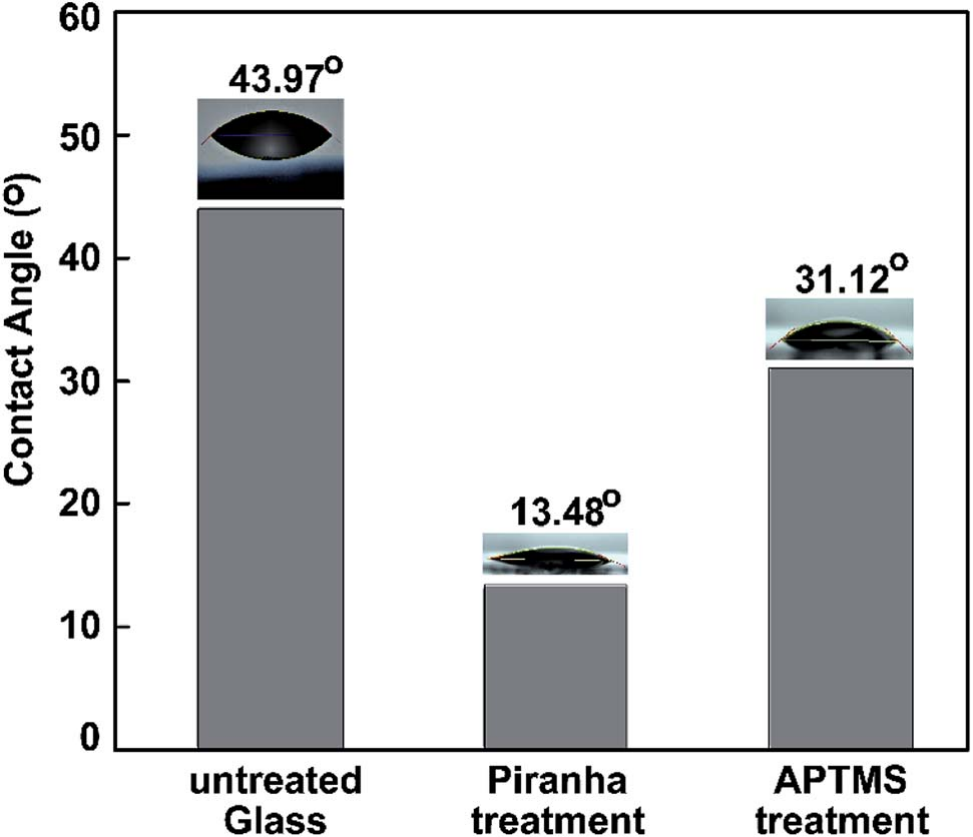
Water contact angles measured on pristine glass, piranha-treated glass, and APTMS-modified glass.

Water contact angle (WCA) measurements were performed to study the surface modification. We measured the water contact angles to characterize the surface of the bare glass substrate after each step of functionalization. Three different types of surface wetting properties, based on the untreated glass, piranha-treated glass, and ATPMS-modified glass, can be observed in Fig. 7.

After surface activation, the contact angle immediately dropped from 43.97° to 13.48° for the piranha-treated bare glass. Piranha causes the removal of contaminants, the generation of –OH groups, and, hence, the hydrophilization of the surface.^70^ The drastic decrease in contact angle (by 30.49°) exhibited that the hydrophobic coating of the as-prepared glass slide became hydrophilic after 10 min of piranha treatment etching, owing to the surface being left with H-terminals on top of the glass substrate. After immersing the piranha-activated glass in aqueous APTMS solution for 2 h, we observed a significant increase in the contact angle to 31.12°. These results can be explained based on the formation of a silane layer on the substrate terminated with free hydrophilic amine groups. The results confirm the reduction of the hydrophilic surface modification following amine treatment with ATPMS.^71,72^ The dynamic WCA measurements were in agreement with the above-mentioned FT-IR investigations.

### Controlling the embedding time for the self-organization of Au NPs on glass

**Fig. 8:**
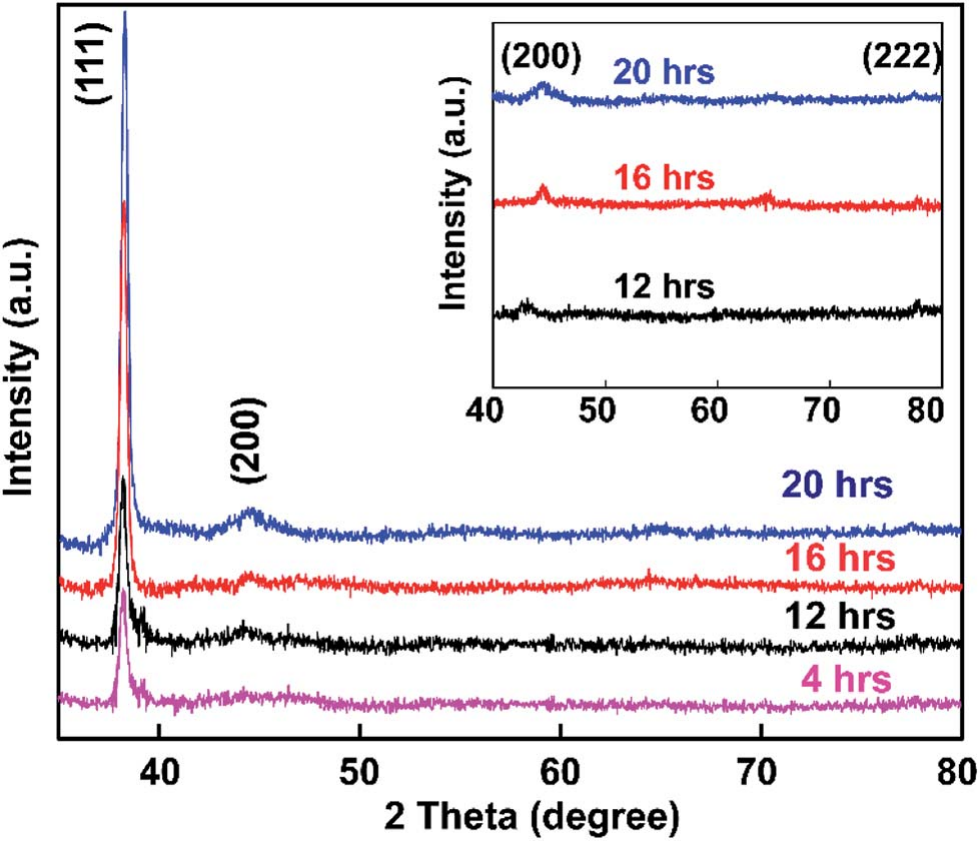
Scattering intensity of Au NPs on a glass substrate as a function of embedding time from 4–20 h. The inset shows the XRD patterns of Au NPs embedded for 12, 16, and 20 h over the diffraction range from 40° to 80°.

The synthesized colloidal Au NP solution was exposed to glass based on soaking times of 4, 12, 16, and 20 h, and samples were then subjected to powder XRD analysis (Fig. 8).

Fig. 8 shows the spectra of APTMS-functionalized glass substrate after immersion in Au NPs solution. XRD patterns of the synthesized Au NPs displayed Bragg reflections representative of the face-centered cubic (FCC) structure of gold.^73^ The intensity of the (111) peak at 37.3° was much stronger than those of the (200), (220), and (222) peaks at 43.3°, 62.9°, and 79.5° respectively (inset of Fig. 8). The peak intensities of the samples increased with increasing Au NP embedding time, which indicates that pure Au crystals were obtained using the present method.

Comparing the results of this study with the reference XRD pattern of Au NPs, the intense peak at 37.3° represents preferential growth in the (111) direction. No other diffraction peaks or impurities were found, verifying that the as-prepared sample contains highly purified Au NPs.

Also, the average crystallite size of the Au NPs was estimated through the well-known Debye–Scherrer equation upon determining the width of the (111) Bragg reflection using the formula *d* = *kλ*/*β* cos *θ*, where *d* is the grain size, *k* is the Scherrer constant with a value of 0.9, *λ* is the wavelength of the X-ray radiation used, *β* is the full width at half maximum (FWHM), and *θ* is the Bragg angle.^74^ From the Scherrer formula, the average crystallite size of the Au NPs obtained after 4, 16, 12, and 20 h is 20.4 nm. Thus, the XRD patterns clearly demonstrate that the Au NPs were attached to the glass surface.

**Fig. 9:**
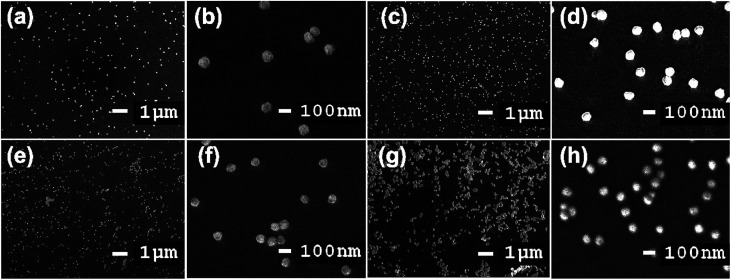
SEM images of glass-piranha-APTMS after soaking in Au NP solution for (a) and (b) 4 h, (c) and (d) 12 h, (e) and (f) 16 h, and (g) and (h) 20 h. The scale bars are 1 μm and 100 nm.

The size, orientation, and shape of the prepared Au NPs were further studied *via* SEM, and typical images are reported (Fig. 9). SEM micrographs of the samples show the surface coverage and dispersion of the Au NPs on the functionalized surfaces. A major determinant of the optical properties of Au NPs is their shape. The morphology of the Au NPs is an isotropic sphere, and they appear as a brown powder. Spherical Au NPs are ideal for use in applications such as drug delivery, tumor therapeutics, cancer diagnosis and treatment, and lateral flow rapid tests. These results showed the strong relationship between the plasmonic properties and morphologies of the metallic nanoparticles. Differences in the glass immersion time in the gold nanoparticle solution affect the dispersion of nanoparticles on the surface of the material. Particularly, after a short period of immersion time (4 h), there were hardly any self-aligned Au NP arrays on the glass substrate. This result clearly indicated that the immersion time was of importance, and it was extended to 12, 16, and 20 h. Fig. 9c suggests that obtaining a substrate with favorable homogeneity required an appropriate immersion time of 12 h. The results (Fig. 9e–g) also suggested that it was hard to form a uniform Au nanofilm *via* only extending the immersion time to 16 or 20 h. The uniformity and stacking density of the Au NPs obviously improved. The immersion time seemingly influenced the monodispersity of the Au NPs and the mean interparticle gaps on the substrate.

High-magnification SEM images of the substrate are shown in Fig. 9b, d, f and h. The average size of the Au NPs prepared *via* employing gold nanoseed activation and with immersion times of 4, 12, 16, and 20 h was approximately 84 nm. The Au NPs are immobilized on the substrate but only bound loosely to the surface through weak van der Waals interactions at play in the Au–S system. During synthesis, the Au NP growth process is assisted by adding citrate, which is a stabilizing, and capping agent that can control the morphology and shape of nanoparticles and prevent the aggregation of Au NPs through electrostatic repulsion.

Analyzing the data from XRD and SEM shows that the most appropriate Au NP immersion time on the modified glass substrate is 12 h. The obtained XRD peak collected from the 12 h candidate and its particle homogeneity with the required size captured by SEM arguably make it the most worthwhile sample for further applications.

### SERS analysis of the self-assembled Au NP arrays

To operationalize rapid diagnostic tests, SERS measurements were carried out using Raman scattering on a reflective capillary tube and an as-prepared Au substrate analyte with the same volume. We use these label-free SERS sensors for detecting a neurotransmitter present at low concentrations. Fluorescent serotonin was used as a Raman probe for the evaluation of the SERS activities of these Au NPs at different concentrations. To investigate the stability and reproducibility of the as-prepared Au NPs, SERS spectra of serotonin were obtained on 10 randomly selected samples for comparison.

**Fig. 10:**
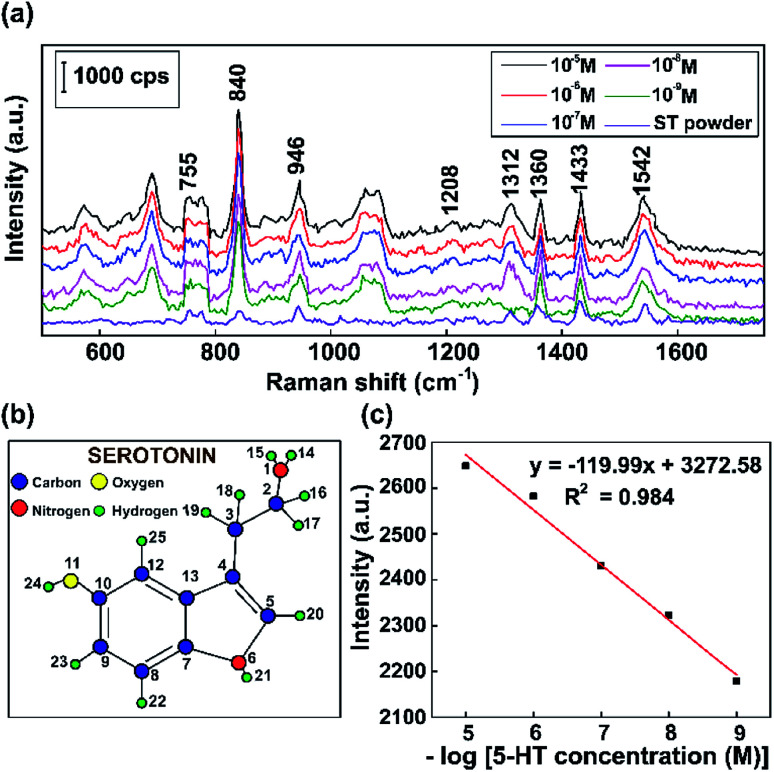
(a) The Raman spectra of serotonin (5-hydroxytryptamine, 5-HT) at different concentrations and a standard 5-HT neurotransmitter (ST powder). (b) The optimized geometry of serotonin. (c) The standard curve between the Raman intensity of the peak at 830 cm^−1^ and the logarithm of 5-HT concentration.

Fig. 10 shows the typical Raman vibration pathways of a standard 5-HT neurotransmitter (5-HT powder) and presents an evaluation of the intensities of characteristic Raman vibrational lines at concentrations ranging from 10^−5^ M to 10^−9^ M 5-HT on SERS substrates based on Au NPs. Fig. 10a shows that some dominant bands that are observed in the Raman spectrum of the standard completely disappear or shift in the SERS spectra of 5-HT.^75^ The cause of the spectral shifts is that the indole NH band shifts downward following the formation of a hydrogen bond with negatively charged gold colloids.^76^ Specific to the SERS spectrum, the ring H^23^–C^9^, H^22^–C^8^, and H^20^–C^5^ out-of-plane rocking vibrations at around 840 cm^−1^, the twisting deformation of H^16^–C^2^–H^17^ at around 1360 cm^−1^, the wagging deformation of H^16^–C^2^–H^17^ and H^18^–C^3^–H^19^ at about 1433 cm^−1^, and the scissoring deformation of H^16^–C^2^–H^17^ at around 1582 cm^−1^ have stronger vibrations than the standard serotonin powder.^77,78^ The spectrum of serotonin powder was analyzed for the confirmation of 5-HT detection using our substrate *via* Raman spectroscopy.^79^ The specific fluctuations of serotonin found in this paper are shown in Table 1.

**Table 1:** The assignment of modes based on wavenumber

Wavenumber (cm^−1^)	Approximate description				
755	C^8^–H^22^	Indole ring	Strong	In-plane	Stretch
	C^12^–H^25^		Medium	In-plane	Stretch
	C^5^–H^20^				
	N^6^–H^21^			In-plane	Stretch
840	H^23^–C^9^	Ring	Strong		
	H^22^–C^8^				
	H^20^–C^5^	Rock		Out-of-plane	
946	H^22^–C^8^	Ring	Strong		
	H^23^–C^9^	Rock		Out-of-plane	
1208	H^24^–O^11^	Rock	Strong	In-plane	
	H^22^–C^8^–H^23^–C^9^	Scissor		In-plane	
1312	C^3^–H^19^–H^18^	Twist	Strong		
	H^16^–C^2^–H^17^	Twist			
	H^20^–C^5^	Rock		In-plane	
1360	H^16^–C^2^–H^17^	Twist	Strong		
	H^20^–C^5^	Rock		In-plane	
1433	H^16^–C^2^–H^17^	Wag	Strong		
	H^18^–C^3^–H^19^	Wag	Medium		
1542	H^16^–C^2^–H^17^	Scissor	Strong		

As shown in Fig. 10c, the results demonstrate that the intensity of the 840 cm^−1^ peak of 5-HT decreases when the concentration was decreased. The overall results are consistent with a localized surface plasmon model, which was created by internalized Au NPs that were excited by the Raman source. The standard curve between the Raman intensity of the 840 cm^−1^ peak and logarithm of 5-HT concentration has a correlation coefficient of 0.984, indicating that this analysis provides high reliability for the qualitative characterization of the 5-HT neurotransmitter. Moreover, by performing SERS experiments with Au NPs, concentrations of 5-HT as low as 10^−9^ M can be detected. The results clearly show that the Au NP platform for SERS substrates has potential sensitivity and high performance, making it suitable for the diagnosis of numerous neurological diseases, particularly those related to the 5-HT neurotransmitter.

For the further goal of detecting 5-HT in biological fluids, serotonin was mixed with dopamine (DA) in the presence of ascorbic acid to confirm the selectivity of our substrate for 5-HT. Dopamine and ascorbic acid were chosen because they coexist with 5-HT in biological fluids, such as in the central nervous system. Dopamine and serotonin are neurotransmitters in the brain and the central nervous system (CNS) that have important roles in regulating mood and emotion and other functions. Also, samples used to detect 5-HT are likely to be mixed with ascorbic acid because this substance is abundant in the intestines, especially the small intestine. The concentrations of 5-HT, dopamine, and ascorbic acid were 10^−7^ M. As shown in Fig. 1S,† the peaks of 5-HT were slightly shifted to 754, 833, 946, 1202, 1308, 1350, 1440, and 1547 cm^−1^. The additional peaks at 1370, 1479, and 1622 cm^−1^ belong to dopamine.^80^ There were no peaks from ascorbic acid due to its lack of affinity for Au.^81^ In general, 5-HT can be detected by our SERS substrate in a complex mixture. A schematic diagram showing the direct APTES [(3-aminopropyl)triethoxysilane] functionalization of Au NPs is shown in Fig. 2S.† The surface chemistry includes amines to immobilize Au NPs *via* covalent attachment to the Au NPs. For SERS detection, we immersed the Au NP-coated glass in serotonin; one –NH group attached to one Au NP and the opposite –NH attached to a second Au NP, forming a dimer.

In order to confirm the stability and reproducibility of our substrate, the Raman analysis of 5-HT (10^−8^ M) was carried out. As shown in Fig. 3S,† the Au NPs were smooth and uniform. The Raman spectra were clear with distinct peaks. This indicated the high performance of our substrate for 5-HT detection. To confirm the long life of our solution, Raman spectra of 5-HT (10^−9^ M) adsorbed onto the substrate were collected after 30 and 60 days, and they are shown in Fig. 4S.† After a long storage time, there was no dramatic decrease of 5-HT intensity. Both resulting spectra are almost identical in terms of both the positions of the Raman shifts and the peak intensities. This implied that our substrate can be stable for weeks without any issues, and its subsequent use for SERS still yields the same results. It took about 1 hour and a half to measure one sample using our SERS substrate. This is the advantage of our material, allowing for the quick testing of any sample.

## Conclusions

Based on the results, an LSPR-based sensor using Au NPs demonstrated great spectral shift characteristics due to its unique optical properties. We, for the first time, synthesized Au NPs *via* a simple, inexpensive, and rapid ultrasonic bath method (40 min). The time-dependent LSPR extinction in synthesized Au NP solutions was studied *via* UV-vis spectra, exhibiting characteristic bands centered at 527.6 nm. The crystalline structure, size, shape, and distribution of Au NPs on the glass sensors were confirmed *via* XRD and SEM. LSPR can be tailored *via* designing plasmon-enhanced nanostructures with different sizes and shapes, and adjusting the components of the materials provides a great opportunity to develop clinical lab applications and tremendous ultra-sensitive chemical and biological sensors. We found that the obtained composites exhibited characteristics suitable for LSPR sensor use and applications both in biology and technology (*e.g.* photonics) due to the unique optical properties. The plasmon excitation band of a single Au NP plasmon mode can be clearly observed in all the samples, confirming the formation of Au NPs. The SERS activities of these Au NPs were evaluated using serotonin as a Raman probe. The SERS enhancement effect was shown and ultrasensitive serotonin detection was obtained on Au NPs at concentrations as low as 10^−9^ M. These capabilities not only show promising research potential for rapid serotonin detection but they can also be extended to other forms of biosensors.

## Conflicts of interest

There are no conflicts to declare.

## Supplementary Material

RA-010-D0RA05271J-s001
