# Supplementary material for: The highly sensitive determination of serotonin by using gold nanoparticles (Au NPs) with a localized surface plasmon resonance (LSPR) absorption wavelength in the visible region
Source: RSC Adv. 2020 Aug 20;10(51):30858–69. doi: 10.1039/d0ra05271j (PMC9056339; doi:10.1039/d0ra05271j)
Supplement: RA-010-D0RA05271J-s001 [file RA-010-D0RA05271J-s001.pdf]

Electronic Supplementary Material (ESI) for RSC Advances.
This journal is © The Royal Society of Chemistry 2020

Supplementary Material

For

Highly sensitive determination of serotonin by using gold nanoparticles (Au NPs) with localized surface plasmon resonance (LSPR) absorption wavelength a visible region

Phuong Que Tran Do,[¥a,b] Vu Thi Huong, [¥a,b] Nguyen Tran Truc Phuong,[a,b] Thi-Hiep Nguyen,[b,c] Hanh Kieu Thi Ta,[a,b] Heongkyu Ju,[d] Thang Bach Phan,[b,e,f] Viet-Duc Phung,[g,h] Kieu The Loan Trinh[i] and Nhu Hoa Thi Tran[*a,b]

[a.] *Faculty of Materials Science and Technology, University of Science, HoChiMinh City, Viet Nam*

[b.] *Vietnam National University, HoChiMinh City, Viet Nam*

[c.] *Tissue Engineering and Regenerative Medicine Laboratory, Department of Biomedical Engineering, International University, HoChiMinh City, Viet Nam*

[d.] *Department of Nano-Physics, Gachon University, Seongnam-si, Gyeonggi-do 13120, Republic of Korea*

[e.] *Center for Innovative Materials and Architectures (INOMAR), HoChiMinh City, Viet Nam*

[f.] *Laboratory of Advanced Materials, University of Science, HoChiMinh City, Viet Nam*

[g.] *Future Materials and Devices Laboratory, Institute of Fundamental and Applied Sciences, Duy Tan University, Ho Chi Minh City, 700000, Viet Nam*

[h.] *Faculty of Environmental and Chemical Engineering, Duy Tan University, Da Nang, 550000, Viet Nam*

[i.] *Department of Industrial Environmental Engineering, College of Industrial Environmental Engineering, Gachon University, Seongnam-si, Gyeonggi-do 13120, Republic of Korea*

[¥] P.Q.T.D. and V.T.H. contributed equally to this work.

*Corresponding author: ttnhoa@hcmus.edu.vn

The concentration of 5-HT, dopamine, and ascorbic acid were 10^{-7} M. This sample was absorbed on our SERS substrate and measured by Raman spectroscopy. The result was shown in Fig. 1S. The spectrum of 5-HT (10^{-7} M) was inserted for the comparison. As shown in this figure, the peaks of 5-HT were slightly shifted to 754, 833, 946, 1202, 1308, 1350, 1440, and 1547 cm^{-1}. The additional peaks at 1370, 1479, 1622 cm^{-1} were belonged to dopamine.[80] There was no peak of ascorbic acid because it was nonaffinititiy for Au.[81] In general, 5-HT can be detected by our SERS substrate in complex mixture.

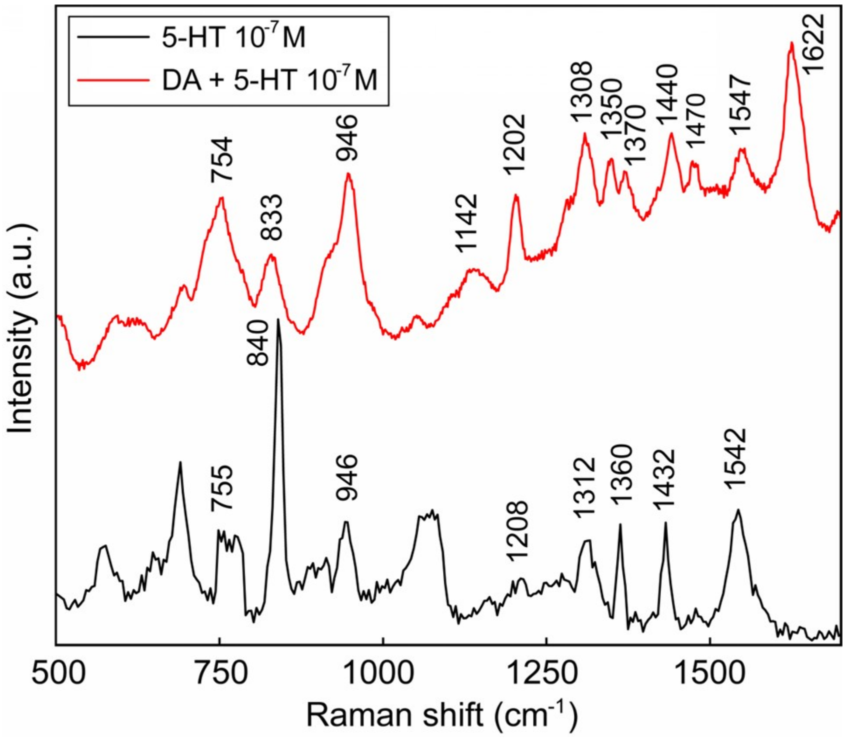

Figure 1S. Raman spectra of 5-HT (10^{-7} M) and the mixture of 5-HT and dopamine in the presence of ascorbic acid.

In comparison with other reports, 5-HT detected by our Au NPs has some advantages. First, the lowest 5-HT's concentration could be detected by our Au NPs was 1000 times lower than the polycrystalline silver electrode prepared by Song et al..[75] The peaks of 5-HT spectra was clearer and sharper than the substrate from graphene-Au nanopyramid heterostructure fabricated by Wang et al..[82] Moreover, the spectra of 5-HT detected by our substrate was more reproducible than the Ag colloids synthesized by Qiu et al..[83] Our substrate was facilely fabricated and high efficient in 5-HT detection with the selective mechanism of Au NPs to amine group of 5-HT.

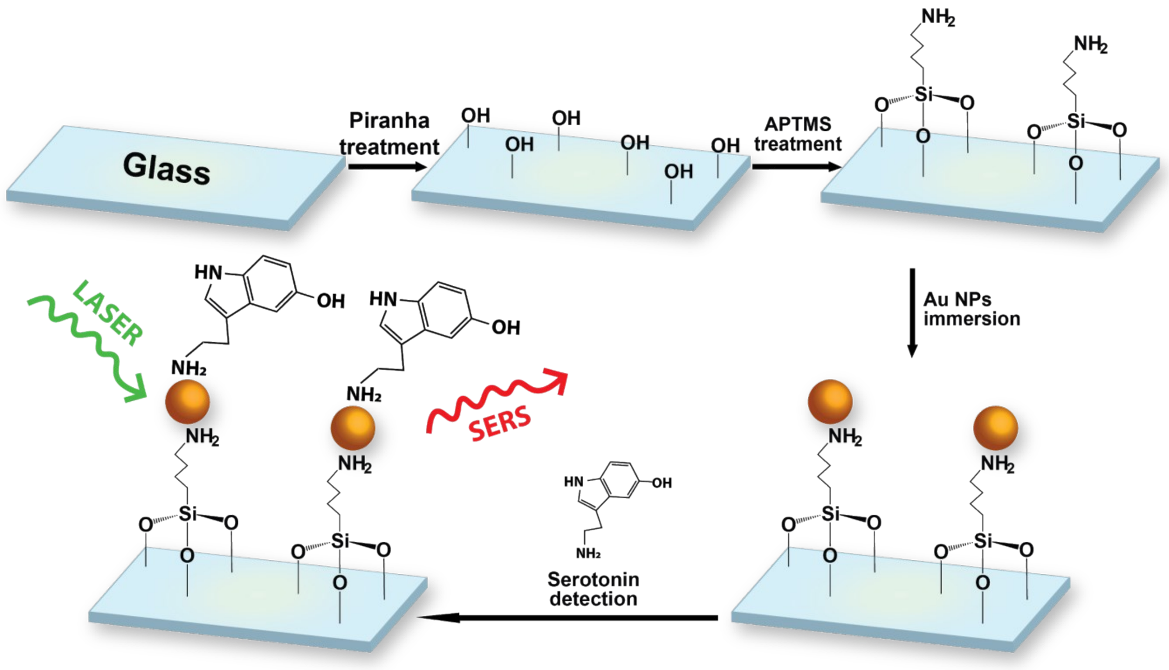

Fig. 2S. Schematic diagram of 5-HT molecules linking with the Au NPs modified on glass substrate.

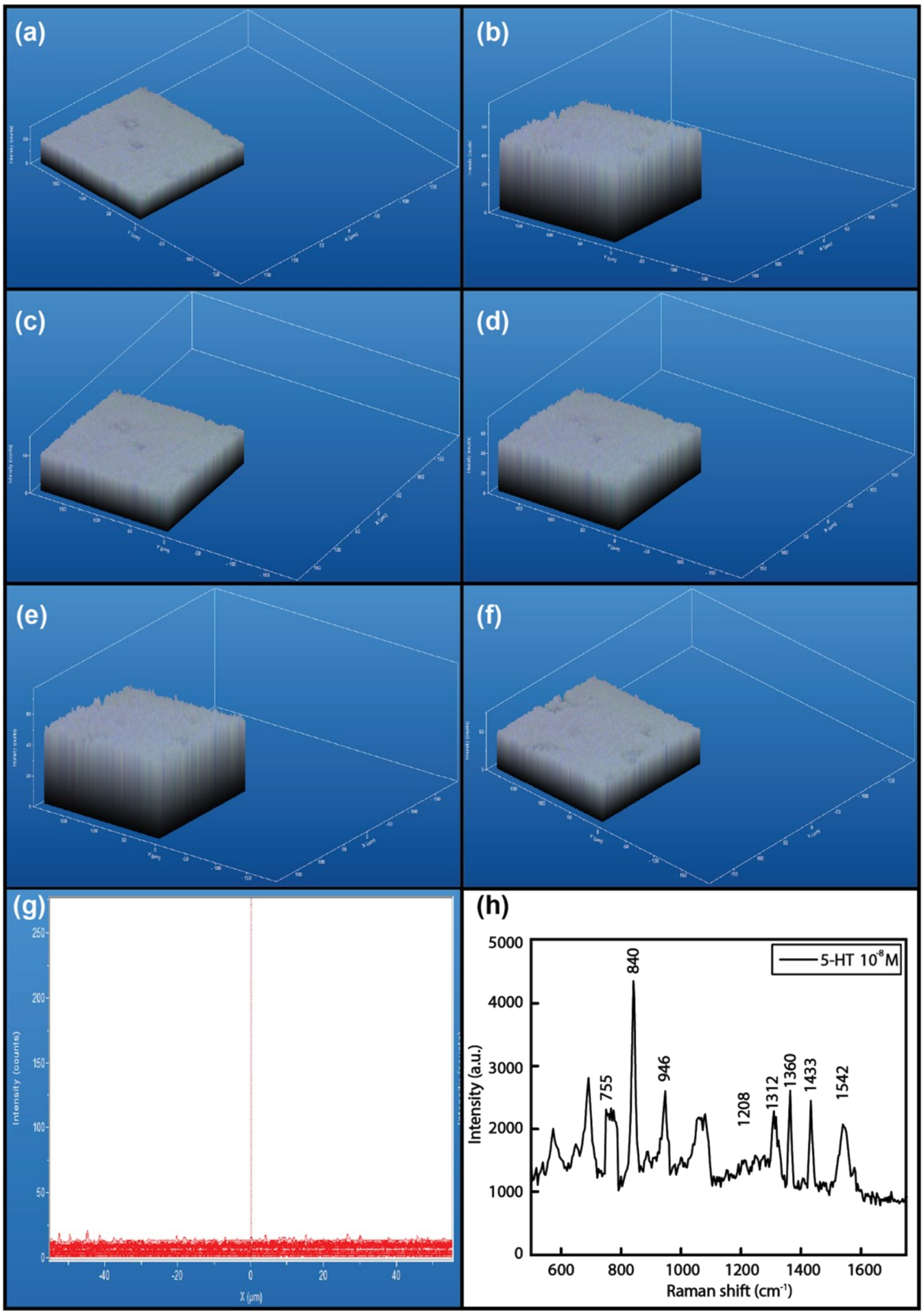

Fig. 3S. Raman mapping of 5-HT (10^{-8} M) adsorbed on our substrate.

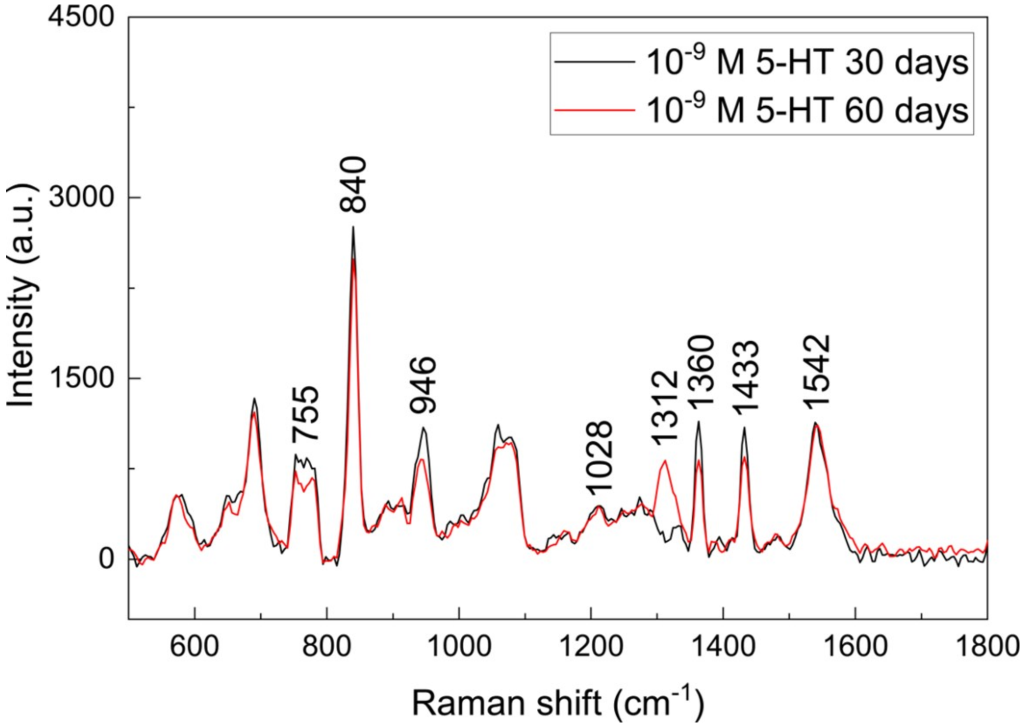

Fig. 4S. Raman spectra of 5-HT at the concentration of 10^{-9} M on Au NPs coated substrate in 30 and 60 days.

References:

75 P. Song, X. Guo, Y. Pan, Y. Wen, Z. Zhang and H. Yang, *J. Electroanal. Chem*, 2013, **688**, 384-391.

80 F. S. Manciu, M. Manciu, J. D. Ciubuc, E. M. Sundin, K. Ochoa, M. Eastman, W. G. Durrer, J. Guerrero, B. Lopez, M. Subedi and K. E. Bennet, Biosensors, 2019, 9, 3.

81 E. Vander Ende, M. R. Bourgeois, A. I. Henry, J. L. Chavez, R. Krabacher, G. C. Schatz and R. P. Van Duyne, *Analytical chemistry*, 2019, **91**, 9554-9562.

82 P. Wang, M. Xia, O. Liang, K. Sun, A. F. Cipriano, T. Schroeder, H. Liu and Y. Xie, *Anal. Chem.*, 2015, **87**, 10255-10261.

83 C. Qiu, K. E. Bennet, J. R. Tomshine, S. Hara , J. D. Ciubuc , U. Schmidt , W. G. Durrer, M. B. McIntosh, M. Eastman and F. S. Manciu, *Biointerface Res. Appl. Chem.*, 2017, **7**, 1921-1926.
